# *Conidiobolus pachyzygosporus* invasive pulmonary infection in a patient with acute myeloid leukemia: case report and review of the literature

**DOI:** 10.1186/s12879-020-05218-w

**Published:** 2020-07-22

**Authors:** E. Stavropoulou, A. T. Coste, C. Beigelman-Aubry, I. Letovanec, O. Spertini, A. Lovis, T. Krueger, R. Burger, P. Y. Bochud, F. Lamoth

**Affiliations:** 1 Infectious Diseases Service, Department of Medicine, Lausanne University Hospital and University of Lausanne, Lausanne, Switzerland; 2 Institute of Microbiology, Department of Laboratories, Lausanne University Hospital and University of Lausanne, Lausanne, Switzerland; 3 Department of Diagnostic and Interventional Radiology, Lausanne University Hospital and University of Lausanne, Lausanne, Switzerland; 4 Institute of Pathology, Lausanne University Hospital and University of Lausanne, Lausanne, Switzerland; 5 Service and Central Laboratory of Hematology, Department of Oncology, Lausanne University hospital and University of Lausanne, Lausanne, Switzerland; 6 Service of pneumology, Department of Medicine, Lausanne University Hospital and University of Lausanne, Lausanne, Switzerland; 7 Thoracic Surgery Service, Department of Surgery and Anesthesiology, Lausanne University Hospital and University of Lausanne, Lausanne, Switzerland

**Keywords:** Enthomophthoramycosis, Entomophthorales, Conidiobolomycolosis, Invasive fungal infections

## Abstract

**Background:**

*Conidiobolus* spp. (mainly *C. coronatus*) are the causal agents of rhino-facial conidiobolomycosis, a limited soft tissue infection, which is essentially observed in immunocompetent individuals from tropical areas. Rare cases of invasive conidiobolomycosis due to *C. coronatus* or other species (*C.incongruus, C.lamprauges*) have been reported in immunocompromised patients. We report here the first case of invasive pulmonary fungal infection due to *Conidiobolus pachyzygosporus* in a Swiss patient with onco-haematologic malignancy.

**Case presentation:**

A 71 year-old female was admitted in a Swiss hospital for induction chemotherapy of acute myeloid leukemia. A chest CT performed during the neutropenic phase identified three well-circumscribed lung lesions consistent with invasive fungal infection, along with a positive 1,3-beta-d-glucan assay in serum. A transbronchial biopsy of the lung lesions revealed large occasionally septate hyphae. A *Conidiobolus* spp. was detected by direct 18S rDNA in the tissue biopsy and subsequently identified at species level as *C. pachyzygosporus* by 28S rDNA sequencing. The infection was cured after isavuconazole therapy, recovery of the immune system and surgical resection of lung lesions.

**Conclusions:**

This is the first description of *C. pachyzygosporus* as human pathogen and second case report of invasive conidiobolomycosis from a European country.

## Background

*Conidiobolus* spp*.* are filamentous fungi, which belong to the phylum *Entomophthoramycota* and are responsible for a human disease called conidiobolomycosis. These fungi are insect parasites and can also be found in soil, decaying vegetation and reptile or amphibian droppings [1, 2]. Although *Conidiobolus* spp. seem to be ubiquitous in the world, conidiobolomycosis is mainly a tropical disease as the fungus needs a high level of humidity (> 95%) for germination and growth. The infection usually consists of a rhinofacial cellulitis, which can lead to chronic facial deformity in immunocompetent hosts [1–4]. Few cases of disseminated infections involving multiple organs (lungs, heart, kidneys, spleen or brain) have been reported in immunocompromised individuals, such as hematologic cancer patients or solid-organ transplant recipients [5–14]. While *Conidiobolus coronatus* represents the main pathogenic species in humans, *C. incongruus* and *C. lamprauges* have also been reported, especially in disseminated infections [2, 5, 7, 9, 13]. We report here a case of *C. pachyzygosporus* invasive infection limited to the lungs in a patient with acute myeloid leukemia, which was acquired in Switzerland.

## Case presentation

**Fig. 1:**
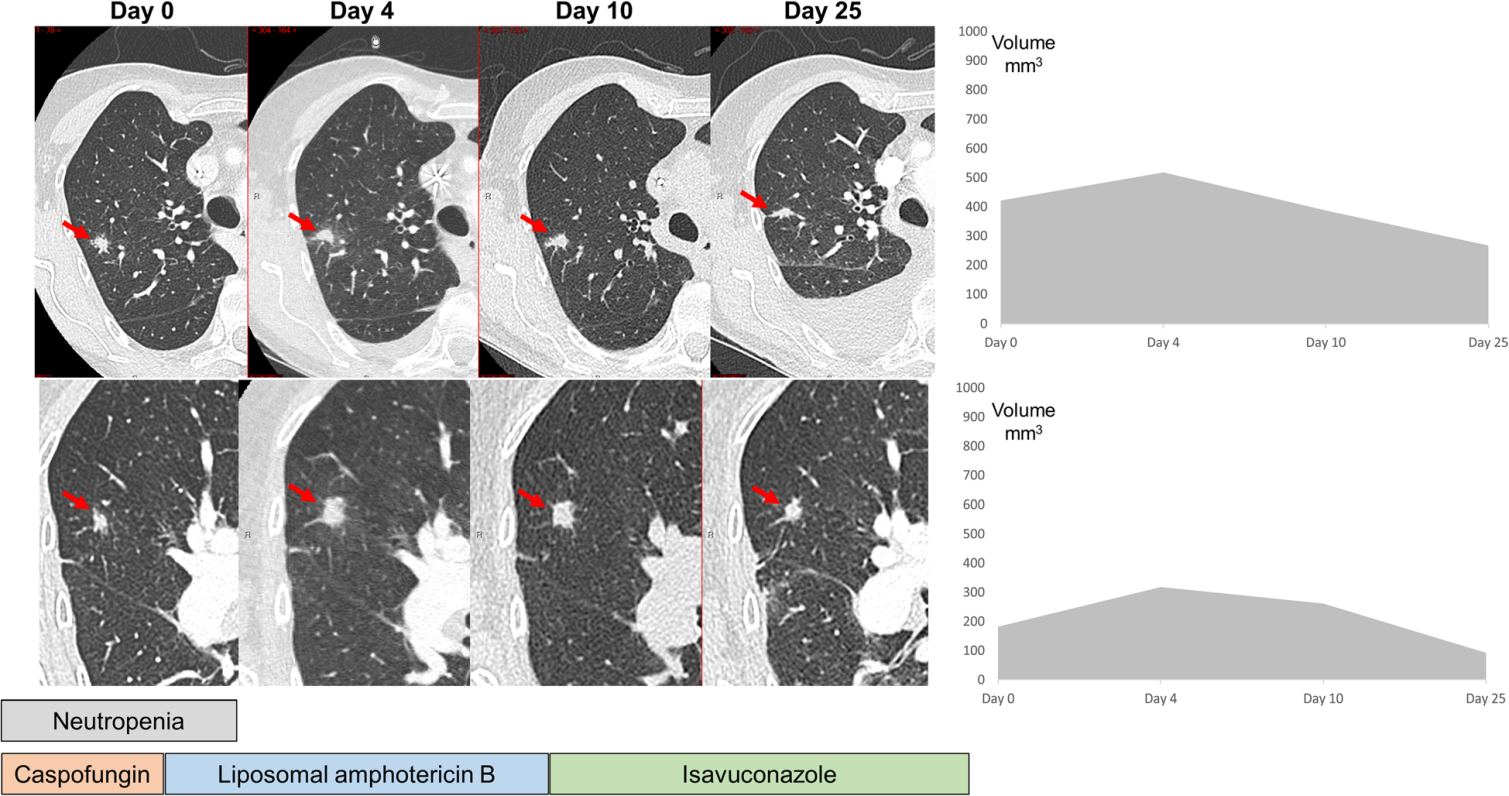
Chest CT imaging at different time points (day 0 = initial diagnosis) showing two nodular lesions in the upper right lobe (red arrows). The graphs in the right panel show volumetric data of the two lesions (volume in mm^3^) at the different time points. Duration of neutropenia (neutrophil count < 500/mm^3^) and sequence of antifungal therapy courses are shown below

**Fig. 2:**
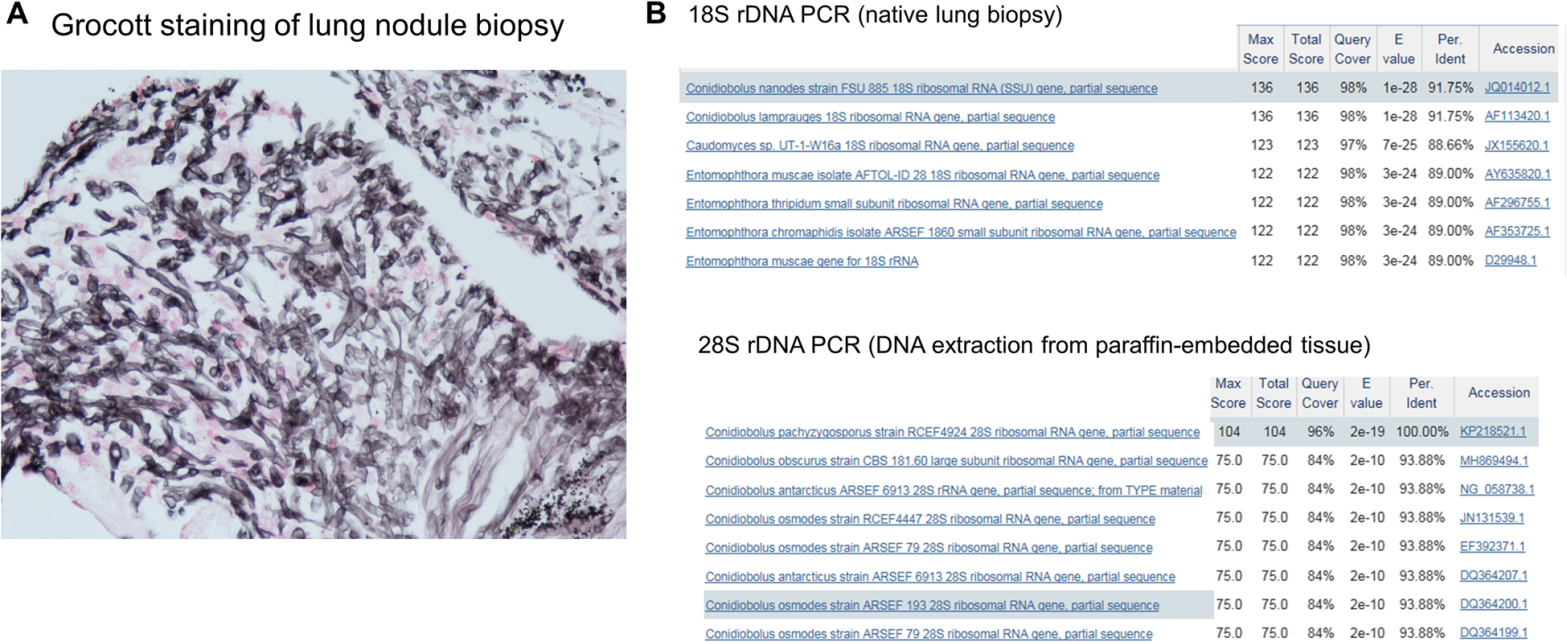
Grocott silver staining of the lung tissue biopsy showing large and tortuous hyphal elements with occasional septa (**a**). Results of PCR analyses performed directly on the lung tissue biopsy (native tissue and following DNA extraction from the paraffin-embedded tissue): 18S rDNA sequencing (upper panel) and 28S rDNA sequencing (lower panel). Sequences were matched using the basic local alignment search tool of the National Center for Biotechnology Information (NCBI) (**b**)

A 71 year-old female of Swiss origin was admitted at the University Hospital of Lausanne (Switzerland) for a diagnosis of acute myeloid leukemia. She underwent induction chemotherapy (cytarabine 200 mg/m^2^ days 1–7 and daunorubicin 60 mg/m^2^ days 1,2 and 3, followed by imatinib and then dasatinib 140 mg qd from day 3) and triple intrathecal chemotherapy (cytarabine, methotrexate, hydrocortisone). Chemotherapy-induced neutropenia (i.e. neutrophil count < 500/mm^3^) occurred 8 days later and antifungal prophylaxis with fluconazole (400 mg qd) was started. On the same day, the patient developed febrile neutropenia due to *Streptococcus mitis* bacteremia and was treated with piperacillin-tazobactam. The patient had persistent neutropenic fever despite broad-spectrum antibiotic therapy. Meanwhile, the monitoring of 1,3-d-beta-glucan (BDG) in serum (Fungitell™, Associates of Cape Cod, MA) performed twice weekly was positive on two consecutive values (213 and 104 pg/ml on day 5 and 8 of neutropenia, respectively). The galactomannan testing in serum was negative. Fluconazole was then switched to caspofungin (70 mg on day 1, followed by 50 mg qd). A chest and abdominal computerized tomography (CT) performed on day 9 of neutropenia revealed two well-circumscribed opacities in the right superior lobe and another smaller nodule in the right inferior lobe (Fig. 1). Caspofungin was switched to liposomal amphotericin B (5 mg/kg qd). The BDG test turned negative 3 days after the start of amphotericin B therapy. The patient recovered from neutropenia after 11 days and a bronchoscopy with broncho-alveolar lavage (BAL) was performed on the same day. Cultures and galactomannan testing were negative in BAL fluid. *Aspergillus fumigatus*-specific PCR and 18S rDNA panfungal PCR, as previously described [15], were also negative on this sample. Because of worsening dyspnea, another CT was repeated 4 days after the initial imaging, which showed an increase in size of the lung lesions with appearance of ground-glass opacity (Fig. 1). Liposomal amphotericin B was switched to oral isavuconazole (200 mg tid on day 1 and 2, followed by 200 mg qd) because of renal failure potentially attributed to amphotericin B, and a bronchoscopy with radial ultrasound assisted transbronchial biopsies was performed, as previously described [16]. Histologic examination of the lung biopsy showed bronchopulmonary parenchyma with necrosis and presence of large and tortuous mycelia with occasional septa at Grocott and periodic-acid-Schiff (PAS) staining (Fig. 2a). Cultures and *A. fumigatus*-specific PCR performed on the native lung tissue biopsy were negative, but the 18S rDNA panfungal PCR was positive at 1582 copies/ml for a *Conidiobolus* spp. with the highest score for *C. nanodes*/*lamprauges* (Fig. 2b). In order to confirm this result, DNA was extracted from the histopathologic paraffin-embedded tissue showing the mycelial elements. The 18S rDNA PCR was positive for a mold belonging to the order *Entomophthorales*, but discrimination at genus/species level was not possible. PCR targeting the 28S rDNA, which has demonstrated good discrimination for identification of *Conidiobolus* spp. at species level [17], was performed on this sample and provided an optimal score with 100% identity for *Conidiobolus pachyzygosporus* (Fig. 2b).

Further patient history revealed no particular exposition within the last 2 years, except a travel in Madeira 3 months earlier. Abdominal and cranial CT did not show evidence of dissemination to other organs. Isavuconazole therapy was continued with trough plasma concentrations within the expected therapeutic range (3.8 mg/l at day 7 of therapy). Serial chest CTs performed on day 10 and 25 of antifungal therapy showed a significant regression of the size of the lung lesions (Fig. 1). The patient was in oncological remission following induction chemotherapy and tyrosine kinase inhibitors (ponatinib, then nilotinib) were maintained awaiting an allogeneic hematopoietic stem cell transplantation. Considering the high immunosuppressive risk associated with this procedure, it was decided to surgically remove the residual lung lesions. Wedge resections of the three nodules were performed. Isavuconazole was interrupted 1 week later after a total of 2 months of antifungal therapy, because of the development of hepatic test disturbances of probable toxic origin. A chest CT performed 2 months after interruption of antifungal therapy did not show any sign of recurrent disease.

## Discussion and conclusions

We describe here a case of proven invasive fungal infection attributed to *Conidiobolus pachyzygosporus* in a patient with acute myeloid leukemia. Although the mold was not isolated in cultures, the histopathologic description of large hyphae evocative of a zygomycetous mold despite the presence of occasional septa is consistent with previous histological descriptions of *Entomophthorales* in human tissues [2, 8, 9, 12, 13]. Direct molecular testing performed by 18S rDNA PCR on both the native tissue biopsy and the DNA extracted from the paraffin-embedded tissue led to the identification of a *Conidiobolus* spp. Identification at species level was achieved by 28S rDNA sequencing with a highest score and unique match with 100% identity for *Conidiobolus pachyzygosporus*. While 18S rDNA sequencing lacks discrimination for species identification among fungi of the order *Entomophthorales*, 28S rDNA amplification was shown to be able to discriminate species among the genus *Conidiobulus*, including *C. pachyzygosporus* [17]. This novel species has been described for the first time in 2018 in samples of plant detritus from China and has never been associated with infections in humans up to now [17]. Our literature search identified only 10 cases of invasive *Conidiobolus* infections including the current one (Table 1). Most cases were disseminated infections involving multiple organs and six of them were observed in patients with hematologic malignancies. Mortality was high (about 70%).

**Table 1:** Case reports of invasive fungal infections due to *Conidiobolus* spp.

**Year of publication, region/country (reference)**	**Underlying conditions**	**Organs affected**	**Species**	**Treatment (dose), duration and outcome**
1970, West Virginia (USA) [7, 10]	1 year-old male, no underlying conditions	Mediastinum, lungs, pericardium	*C. incongruus*	Deoxycholate amphotericin B (1 mg/kg/day), 10 weeks Outcome: cure
1983, Thailand [5]	20 year-old female, no underlying conditions	Soft tissues (breast), lungs, mediastinum, liver, gastro-intestinal tract	*C. incongruus*	Co-trimoxazole (2 g/day), duration NS Outcome: death
1990, Texas (USA) [8]	29 year-old male, cocaine abuse	Endocardium, blood, skin, heart, lungs, kidneys, brain, muscles	*Conidiobolus* spp.	None Outcome: death
1992, Mississippi (USA) [12]	64 year-old male, kidney transplantation	Lungs, myocardium, brain, kidney, thyroid	*C. coronatus*	Deoxycholate amphotericin B (50 mg every other day), until death Outcome: death
1994, Maryland (USA) [13]	32 year-old female, lymphocytic lymphoma with leukemic transformation (neutropenia)	Lungs, pericardium	*C. incongruus*	Deoxycholate amphotericin B (0.5 mg/kg/day, then 1.5 mg/kg/day) and flucytosine (150 mg/kg/day), until death Surgery Outcome: death
2009, India [11]	10 year-old female, T-cell acute lymphoblastic leukemia	Sinus, soft tissues (facial)	*C. coronatus*	Amphotericin B (NS), until death Surgery Outcome: death
2010, Germany [14]	78 year-old female, myelodysplastic syndrome	Sinus, soft tissues (facial), brain	*C. incongruus*	Liposomal amphotericin B (200 mg/day), until death Surgery Outcome: death
2011, Japan [9]	61 year-old male, mantle cell lymphoma, allogeneic HSCT	Lungs, heart, spleen, kidney, bladder, thyroid	*C. lamprauges*	Micafungin (150 mg/day) and liposomal amphotericin B (2.5 mg/kg/day), then intravenous voriconazole (6 mg/kg/day on day 1, then 4 mg/kg/day) and micafungin (150 mg/day), until death Outcome: death
2018, Wisconsin (USA) [6]	15 year-old male, B-cell lymphoblastic leukemia (neutropenia)	Sinus, lungs	*C. coronatus*	Liposomal amphotericin B (10 mg/kg/day) and anidulafungin (1.5 mg/kg/day) and oral terbinafine (250 mg twice per day), duration NS Surgery, granulocyte transfusion Outcome: cure
2019, Switzerland (present case)	71 year-old, acute myeloid leukemia (neutropenia)	Lungs	*Conidiobolus* spp.	Caspofungin (70 mg/day on day 1, then 50 mg/day), then liposomal amphotericin B (5 mg/kg/day), then oral isavuconazole (200 mg three times per day on day 1 and 2, then 200 mg/day), 2 months Surgery Outcome: cure

*NS* Not specified

Interestingly, the BDG marker in serum was positive in our case. Analyses of the cell wall of *Entomophthorales* suggest the presence of significant amount of 1,3-d-beta-glucan [2]. Positivity of the BDG marker in serum was previously reported in a case of *C. lamprauges* infection [9], but lack of BDG detection was reported in a case of *C. incongruus* infection [14]. Therefore, data are still scarce to assess the value of the BDG test for diagnosis and follow-up of conidiobolomycosis.

*Conidiobolus* spp. are notoriously resistant in vitro to most antifungal agents, including azole drugs [18, 19]. In the absence of isolation of the mold by conventional cultures, we could not perform antifungal susceptibility testing in this case. Therapeutic options were limited by the previous nephrotoxicity attributed to liposomal amphotericin B and isavuconazole was continued because of favorable clinical and radiological evolution. Significant reduction of the lung lesions was observed which could be attributed to the antifungal effect of isavuconazole and/or neutrophil recovery. Finally, complete surgical removal of the lung lesions was performed without relapsing disease in follow-up. Our case highlights the importance of multilayered therapeutic approaches combining surgery, granulocyte transfusion and antifungal therapy, as previously suggested for invasive *Conidiobolus* infections [6].

While conidiobolomycosis is classically described as a tropical disease, the rare cases of invasive infections in hematologic cancer patients have been described in temperate regions (USA, Japan, Europe). For most of them, the causal agent was a *Conidiobolus* spp. other than *C. coronatus*, (the main cause of rhinofacial conidiobolomycosis in tropical areas) [6, 9, 13, 14]. While *Conidiobolus* spp. are supposedly ubiquitous and have even been isolated in environmental samples of northern European countries [20], detection of *Conidiobolus* spp. from clinical specimens has rarely been reported in Europe [14, 21]. The fact that *Conidiobolus* spp. require a high level of humidity for their growth and development could explain why their pathogenicity is usually limited to tropical areas [2]. Whether climate changes and global warming may alter the epidemiology of invasive fungal infections in the future, with emergence of tropical fungi in temperate regions, remains an open question. It is noteworthy that the present case occurred during a summer period, when an unusual heat wave took place in Europe, including in Switzerland. Increasing use of molecular tools may also impact fungal epidemiology by revealing novel fungal pathogens that could be missed by conventional culture methods.

## Data Availability

The dataset of this case report is available upon reasonable request of the editors and with respect of the confidential rules of our institution and anonymity of the patient.

## Abbreviations

CT: Computed tomography
BAL: Broncho-alveolar lavage fluid
BDG: 1,3-Beta-d-glucan
